# ORBDA: An *open*EHR benchmark dataset for performance assessment of electronic health record servers

**DOI:** 10.1371/journal.pone.0190028

**Published:** 2018-01-02

**Authors:** Douglas Teodoro, Erik Sundvall, Mario João Junior, Patrick Ruch, Sergio Miranda Freire

**Affiliations:** 1 Departamento de Tecnologia da Informação e Educação em Saúde, Universidade do Estado do Rio de Janeiro, Rio de Janeiro, Brazil; 2 SIB Text Mining, Swiss Institute of Bioinformatics, Geneva, Switzerland; 3 Department of Information Science, HEG-Geneva, HES-SO, Geneva, Switzerland; 4 Department of Biomedical Engineering, Linköping University, Linköping, Sweden; 5 Region Östergötland, Linköping, Sweden; Indraprastha Institute of Information Technology, INDIA

## Abstract

The *open*EHR specifications are designed to support implementation of flexible and interoperable Electronic Health Record (EHR) systems. Despite the increasing number of solutions based on the *open*EHR specifications, it is difficult to find publicly available healthcare datasets in the *open*EHR format that can be used to test, compare and validate different data persistence mechanisms for *open*EHR. To foster research on *open*EHR servers, we present the ***o****pen*EH**R B**enchmark **Da**taset, ORBDA, a very large healthcare benchmark dataset encoded using the *open*EHR formalism. To construct ORBDA, we extracted and cleaned a de-identified dataset from the Brazilian National Healthcare System (SUS) containing hospitalisation and high complexity procedures information and formalised it using a set of *open*EHR archetypes and templates. Then, we implemented a tool to enrich the raw relational data and convert it into the *open*EHR model using the *open*EHR Java reference model library. The ORBDA dataset is available in *composition*, *versioned composition* and *EHR open*EHR representations in XML and JSON formats. In total, the dataset contains more than 150 million composition records. We describe the dataset and provide means to access it. Additionally, we demonstrate the usage of ORBDA for evaluating inserting throughput and query latency performances of some NoSQL database management systems. We believe that ORBDA is a valuable asset for assessing storage models for *open*EHR-based information systems during the software engineering process. It may also be a suitable component in future standardised benchmarking of available *open*EHR storage platforms.

## Introduction

Electronic Health Records (EHRs) have a potential to improve the healthcare system by supporting continuing, efficient and quality integrated health care [1,2]. Archetype based EHR systems with shared standardised detailed content models would enable healthcare professionals to access patient record information distributed across multiple sites and represented in several different formats, including narrative, structured, coded and multi-media entries, in a semantically homogeneous environment [3,4]. Nevertheless, they have not yet fulfilled their foreseen role in the healthcare workflow and many environmental, organisational, personal, and technical challenges remain regarding sharing patient data in a healthcare setting using EHR systems [5,6].

To overcome the challenges faced by EHR systems and achieve their goals, the literature suggests that reference models, service interface models, domain-specific concept models and terminologies used in EHRs shall be standardised [7]. Several organisations, including the International Organization for Standardization (ISO/TC 215) [8], Health Level 7 (HL7) [9], the European Committee for Standardization (CEN/TC 251) [10], the Clinical Information Modelling Initiative (CIMI) [11], and the *open*EHR Foundation [12], are developing and publishing formal representations of EHR components, APIs and message protocols to address issues related to the process of seamlessly sharing healthcare data. They have proposed, amongst others, reference models, such as the HL7 FHIR [13] and the *open*EHR Reference Model [14], data exchanging protocols, such as the HL7 Clinical Document Architecture [15], and reference terminologies, such as SNOMED CT [16], that are being increasingly adopted to implement interoperable EHR systems and related components.

In particular, many information systems have already been developed to store and manage EHRs using the *open*EHR storage, retrieval and version-handling formalism as core interoperability components [17,18]. In a review [19], Frade *et al*. identified twenty one projects worldwide implementing *open*EHR-based (or its ISO/CEN 13606 sibling model) information systems developed by the healthcare industry, such as Think!EHR [20], OceanEHR [21] and CloudEHRServer [22], and by the academia [23,24]. Systems like LiU EEE [23] and PyEHR [24] are provided as open-source implementations and use NoSQL databases, such as eXist-db, MongoDB and ElasticSearch, to manage *open*EHR records. Usually, these systems need *open*EHR objects represented in XML or JSON formats to handle inserting operations.

Despite the considerable number of EHR systems available based on the *open*EHR specifications, there are not many published comprehensive assessments of these systems. A literature search in this area reveals that almost every single *open*EHR system implemented was evaluated, when at all, for its functional requirements, such as information representativeness and use of external terminologies, and non-functional requirements, such as scalability and performance, using different datasets [17,18,20,24]. For example, a few attempts to provide a more detailed performance evaluation of databases storing *open*EHR-compliant documents were described in [25,26,27,28]. In these works, the authors provide an evaluation of population-wide querying of *open*EHR data stored in different NoSQL database management systems. However, since they use a private patient identified healthcare dataset, it is hard, if not impossible, to reuse their very same data to compare the results with another system and thus extract reliable comparisons. Indeed, there is no public benchmark dataset that can be used to effectively evaluate performance of implementations. Thus, objective and scientifically sound comparisons between techniques for storing and accessing data applying the *open*EHR formalism in general is difficult.

Due to the sensitive nature of healthcare data content, accessing healthcare data for research purposes, although absolutely needed, is often difficult. Clinical datasets are protected and locked behind healthcare information systems by restrictive legislation and data protection policies [29,30]. The process of getting access to such data for research purposes is expensive, labour-intensive and time-consuming, involving hospital ethical boards, whose decisions can take weeks to months. Thus, it is not rare that healthcare information systems have to be tested and validated with synthetic data, which many times fail to capture errors and idiosyncrasies of real operational data. Nevertheless, comprehensive assessment of EHR systems is crucial for the improvement and more widespread adoption of current solutions. To promote research and foster development, and for more rigorous and reliable testing of *open*EHR-based EHR systems, we present the *open*EHR benchmark dataset (ORBDA), a very large healthcare dataset expressed in the *open*EHR formalism. In this paper, we describe the raw healthcare data and our methodology to convert these data into a dataset compliant with the *open*EHR format, and provide means to access the data and tools developed. To the best of our knowledge, ORBDA is the first publicly available dataset based on the *open*EHR specifications for assessing performance of *open*EHR storage systems.

### Ethics statement

This work has been approved by the research ethics committee of the Pedro Ernesto University Hospital–Rio de Janeiro, Brazil (CEP/HUPE–CAAe: 39418314.9.0000.5259).

### The *open*EHR model

**Fig 1:**
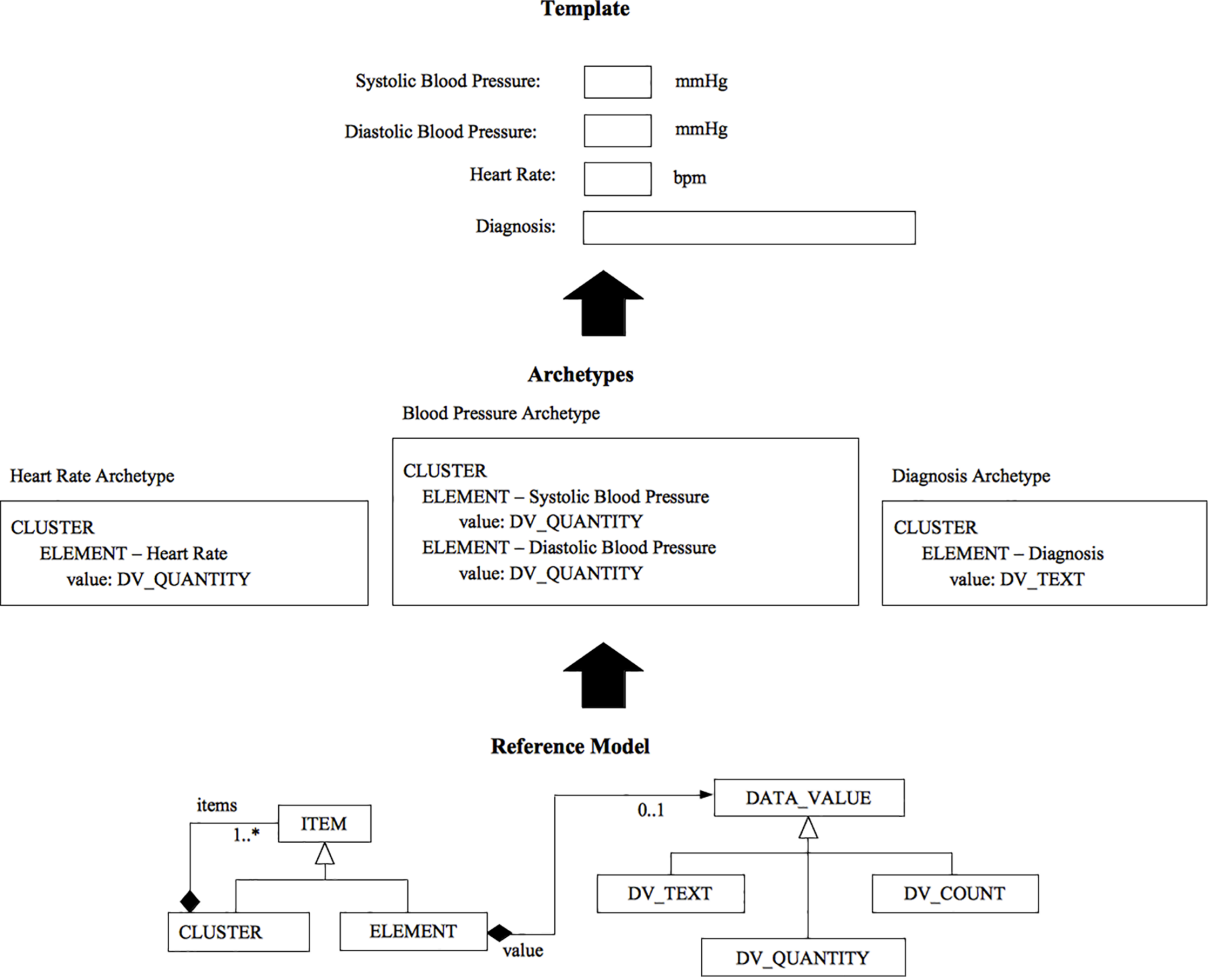
A simplified view of the *open*EHR multilevel model. From the building blocks of the Reference Model, archetypes are created to express domain concepts, which represent valid data structures in the Reference Model. By combining archetypes, we can generate templates that may be used to generate EHR forms, messages and other artefacts. Image credit: Freire *et al*. [27].

The *open*EHR framework aims to allow implementation of future-proof interoperable EHRs by using portable vendor-neutral open models and content definitions. It brings syntactic and semantic interoperability [31,32,33] to the EHR environment using a standardized reference model at the technical level and an archetype model at the clinical knowledge level [34]. To achieve its goals, the *open*EHR framework specifies a multi-level modelling paradigm as showed in Fig 1 [27]. In the first modelling level of the specification, a common reference information model, the *open*EHR Reference Model, defines a set of general reusable building blocks (e.g., data types and structures). These structures are designed to support medico-legal requirements and record management functions, and to ensure that information can be sent and received by systems connected in the EHR network. In a second level, using the Archetype Model, the detailed reusable and domain-specific definitions of healthcare concepts are captured and modelled. This is done using archetypes that, for specific clinical concepts, constrain and define how the Reference Model building blocks are combined, named, and used in tree-like data structures, which provide an information schema [35] for the clinical concept. The archetypes are designed by domain specialists using the Archetype Model usually in online collaboration environments (including discussion/review platforms), such as the Clinical Knowledge Manager (CKM) repository [36]. On a third level, above the archetypes, we have *templates*, which also use the Archetype Model. A *template* is a specification that defines a tree of one or more archetypes, each constraining instances of various reference model types, such as Composition, Section, Entry subtypes and so on. Thus, while there are likely to be archetypes for such things as “biochemistry results” (an Observation archetype) and “SOAP headings” (a Section archetype), templates are used to put archetypes together to form whole Compositions in the EHR, e.g., for “discharge summary”, “antenatal exam” and so on.

## Materials and methods

In this section, we describe the original source relational data and introduce the methods used to represent it in the *open*EHR archetype model. Moreover, we present the methodology used to develop a tool that transforms the source data from a relational model into *open*EHR objects and create the ORBDA dataset. Finally, we describe a proof-of-concept benchmarking experiment that we performed with a subset of the ORBDA dataset containing 10,000 EHRs to demonstrate its application.

### The source data

To create the ORBDA dataset, we used a dataset provided by the Brazilian Public Healthcare System (SUS) through the Department of Informatics of the SUS (DATASUS) database [37,38]. This source dataset contains nation-wide information of pseudo-anonymised patients from healthcare settings distributed across the twenty-seven Brazilian Federation Units. DATASUS systems collect data on the healthcare procedures performed in the Brazilian public health system for the purpose of reimbursement. Data is stored in monthly files and made publicly available through the DATASUS website: http://www2.datasus.gov.br. We used a subset of this data spanning records between January 2008 and December 2012, trying thus to avoid processing data of currently hospitalised patients, as these data are even more sensitive and not relevant for benchmarking purposes.

#### The AIH and APAC datasets

The source dataset used in this work encodes two types of healthcare information, hospitalisation and high complexity procedures, which are available in the Authorisation for Hospital Admission (AIH) and Authorisation for High Complexity Procedures (APAC) databases, respectively. A record in the AIH database is created when a hospital or healthcare unit generates a request for hospitalisation. It aims to validate the pre-admission data and, generally, the diagnosis is reported. Thus, the hospitalisation is not valued immediately, although there is an overall idea of what it will represent as an expense. On the other hand, records in the APAC database are created by providers to register authorised high complexity procedures for the purpose of billing. While AIH records are stored in one single file structure, events registered in the APAC database are subdivided into six different categories–bariatric surgery, chemotherapy, medication, nephrology, radiotherapy and outpatient miscellaneous–and stored on the respective database files. AIH and APAC files are sent electronically to DATASUS by public healthcare providers.

#### Data content

The original records of the AIH and APAC datasets contain administrative, demographic and clinical information, as shown in Table 1, where the core data elements are presented. In addition to these core attributes, each data file has extra attributes that characterises the specific healthcare event that it represents. For example, observations of total urine output over a period and body mass index data elements are stored exclusively in the nephrology and bariatric surgery data files, respectively, while the death indicator data element is present only in the hospitalisation data files. The full version of the data elements available in the AIH and APAC datasets is provided in S1 Table.

**Table 1:** Core data elements of the AIH and APAC datasets.

Data element	Group	Data type
date of discharge	administrative	Date
healthcare unit	administrative	code (CNES)
issue date	administrative	Date
reason for discharge	administrative	code (local)
age	demographic	Numeric
gender	demographic	code (local)
nationality	demographic	code (local)
state	demographic	code (local)
performed procedure	action	code (SIGTAP)
main diagnosis	evaluation	code (ICD 10)
secondary diagnosis	evaluation	code (ICD 10)

To represent the content of the categorical data elements, DATASUS uses a mix of local and standard coding systems. More specifically, three existing coding systems are used: i) ICD 10, the International Disease Classification code, is used to encode diagnoses; ii) CNES, a Brazilian healthcare provider register, is used to encode information about healthcare units; and iii) SIGTAP, a Brazilian procedure, medication, and material terminology, is used to encode performed procedures. For the other categorical data elements, such as gender and reason for encounter, there is a flat definition file, where each code is associated to a label in Portuguese. For example, for gender, the codes F, M and 0 represent the concepts female (*feminino*), male (*masculino*) and not required (*não exigido*), respectively.

#### A dedicated database

To facilitate the extraction of information for a patient, we created a dedicated database to store the AIH and APAC monthly files. In the DATASUS website, the files are originally available in the DBC format. We downloaded DBC files and converted them to the xBase DBF file format using a conversion tool provided by DATASUS. Then, we loaded the DBF files into a PostgreSQL (www.postgresql.org) database using a Java code developed by the authors. Hence, all the records concerning a patient could be retrieved using a single SQL query, avoiding thus searching through all the monthly files for populating a patient’s EHR. For each type of file, i.e., hospitalisation, bariatrics, medication, etc., one dedicated table was created following the same structure of the source data. Since the information in the data files are not normalised, the resulting tables have redundant information, especially for the demographics attributes. To reduce re-identification probabilities, we applied a second level of anonymization by encrypting, truncating or dropping out columns containing HIPAA personal (quasi-)identifiers [29], such as the encrypted patient register number, weight and patient’s ZIP code. Additionally, to make the information more concise to the wider *open*EHR community, very specific DATASUS columns, such as treatment costs, were discarded.

### Design of the *open*EHR compositions and templates

Modelling the source data according to the *open*EHR formalism was performed using the *open*EHR Data Modelling Approach process [39,40]. First, we analysed the DATASUS database and reference models. In the website http://www2.datasus.gov.br, DATASUS maintains a set of files describing the internal structure of the AIH and APAC data files. This information was used to understand the data content and their relations. Second, we classified the attributes into groups of coherent concepts. Third, we reused a set of archetypes provided by the *open*EHR foundation through the CKM repository to represent each group of concepts of the DATASUS dataset [17,26,41]. Whenever the archetypes provided in the CKM were not able to fully cover the data, they were specialised or new ones were developed. The Ocean Informatics Archetype Editor was used for the design, creation and editing of the archetypes. Fourth, we mapped the attributes of the DATASUS relational model to the *open*EHR reference model. Finally, *open*EHR templates were created to represent each table of the DATASUS database, apart from the demographics template, which was derived from specific columns of the source dataset. The Ocean Informatics Template Designer was applied in this stage to create the templates.

### The EHR builder software

After the analysis of the source datasets and some publicly available *open*EHR servers [22,23,24,42], we envisioned three main functional and non-functional requirements for the software to convert the source DATASUS data, available in the relational format, into *open*EHR objects:

- The software shall be able to serialise the source relational data into different file formats and *open*EHR objects. Information systems may store EHRs in different file formats, such as XML and JSON, and they may use different *open*EHR objects as committing units, such as composition and versioned composition.
- The software shall have a high conversion rate performance. The raw dataset contains information from millions of patients. Thus, to have a reasonable conversion time for large scale tests, the software shall be able to convert the source data into the *open*EHR format in the order of few milliseconds per patient.
- The software shall be able to extract the source data from relational databases. This requirement derives from the original conceptual model of the AIH and APAC data files, allied to the need of having records in a single database to facilitate and speed up data access.

The *open*EHR conversion tool, so called SUS-openEHR-Builder, was developed with Java JDK 1.7 and the Java implementation of the *open*EHR reference model (java-libs) [43] was used to model the source data according to the *open*EHR specifications. The tool was tested in a server with the following configuration: Intel^®^ Xeon^®^ CPU E5620 @ 2.40GHz × 16, with 31 GB of memory, running Red Hat Enterprise Linux Server release 5.10 64 bits, with a storage system of 12 TB. We provide the throughput statistics for the reading and writing operations.

### Experiments

To validate the use of ORBDA for assessing persistence of *open*EHR objects, we performed two benchmarking experiments—inserting throughput and retrieval latency—with three NoSQL database management systems: i) Couchbase v4.1, ii) ElasticSearch v2.3 and iii) eXist-db v2.2 [44,45,46]. To insert and retrieve *open*EHR objects from these databases, we developed a Java program that simulates an EHR server client. For insertion operations, the client takes a composition, a versioned composition or an EHR object as input and commits it to one of the database servers. Despite the availability of bulk insertion in the databases, in our experiments we insert objects, i.e., JSON EHRs in Couchbase and ElasticSearch, and XML EHRs in eXist-db, in a serial fashion per client to simulate a production EHR server environment. For the retrieval experiments, we created two types of queries–fetch and search–with 10 different variations. The first query—*fetch*—searches for patient EHRs that match an EHR identifier passed as a parameter and retrieves the composition content of the fetched EHRs. The second query—*search*—searches for compositions that contain a given diagnostic code (ICD) passed as a parameter and retrieves the EHR identifiers of the compositions matching the query.

Three server-client topologies, 1 server– 1 client, 1 server– 8 clients and 3 servers– 8 clients, were tested using a subset of the ORBDA dataset containing 10,000 EHR objects. In the 1 server– 1 client topology, 10,000 EHRs were inserted serially by the client and each retrieval query was submitted 10 times. In the 1 server– 8 clients and 3 servers– 8 clients topologies, 1,250 EHRs were inserted in parallel and each retrieval query was submitted 10 times by each client. The NoSQL databases were deployed in a dedicated server with the following specifications: Intel® Xeon® CPU E5-2670 @ 2.50GHz × 4, with 15 GB of memory and a SSD storage system of 32 GB, running Linux 4.1.10–17.31.amzn1.x86_64 64 bits. We provide statistics for inserting throughput and query latency metrics for the different configurations. Statistical significance is calculated using Wilcoxon test with a 0.95 confidence interval. Results with *P*-value smaller than 0.05 are considered significant.

## Results

In this section, we describe the ORBDA dataset and provide the throughput statistics of the SUS-openEHR-Builder for generating ORBDA datasets in different formats and sizes. In addition, we provide the performance results of the NoSQL databases stressed using ORBDA. All the material developed in this work, including the de-identified ORBDA source datasets, source database model, *open*EHR archetypes and templates, and the SUS-openEHR-Builder code, are publicly available through the project website at www.lampada.uerj.br/orbda.

### ORBDA source database

The source data contains more than 150 million records (AIH: 5.73×10^7^; APAC: 9.59×10^7^) with information from approximately 55.47 million hospitalisation authorisations in the AIH dataset and 7.75 million patients in the APAC dataset, distributed in bariatric surgery (0.07%), chemotherapy (11.66%), medication (59.33%), nephrology (5.07%), outpatient miscellaneous (22.65%), and radiotherapy (1.22%) records. There is no explicit foreign key that connects the hospitalisation (AIH) and high complexity procedure events (APAC) for a given patient. Among the APAC files, there is a link between entities through the national healthcare patient identifier attribute. This information is encrypted (hashed) in the data publicised by DATASUS. Nevertheless, it can still be used to link the APAC records. The patient identifier is not publicised for the AIH table though. In this case, the hospitalisation authorization identifier attribute, which stores a hospitalisation event, is the only explicit attribute that allows us to link records of a same patient within the hospitalisation table. It was taken then as a surrogate for the patient identifier. As shown in Table 2, these records come from more than 4.5 thousand healthcare units distributed across Brazil. They contain more than 12 thousand ICD-10 codes and more than 1.8 thousand procedure codes. Table 2 shows also the characteristics of the dataset at the patient level. However, given the issues with the unique identifiers, they shall be carefully considered. The full ORBDA data source model is provided in S1 Table.

**Table 2:** Statistics of the ORBDA source database content at the dataset and patient levels.

Stats level	Stats item	Attribute	AIH		APAC	
			#	%	#	%
**Dataset**	Patients*	Unique	5.55×10^7^	100.00	7.75×10^6^	100.00
	**Diagnosis**	Unique	1.21×10^4^	100.00	5.09×10^3^	100.00
	**Procedures**	Unique	1.80×10^3^	100.00	7.58×10^2^	100.00
	**Healthcare units**	Unique	4.05×10^3^	100.00	4.61×10^3^	100.00
**Patient**	**Age**	<1	2.10×10^6^	3.78	2.90×10^3^	0.04
		1–4	3.22×10^6^	5.80	1.03×10^5^	1.32
		5–9	1.80×10^6^	3.25	1.71×10^5^	2.20
		10–14	1.30×10^6^	2.34	1.89×10^5^	2.44
		15–19	2.95×10^6^	5.32	2.72×10^5^	3.52
		20–29	9.95×10^6^	17.93	5.80×10^5^	7.49
		30–39	6.85×10^6^	12.36	7.55×10^5^	9.75
		40–49	5.18×10^6^	9.34	1.02×10^6^	13.15
		50–59	4.92×10^6^	8.87	1.37×10^6^	17.65
		60–69	4.58×10^6^	8.25	1.50×10^6^	19.34
		70–79	3.92×10^6^	7.06	1.27×10^6^	16.45
		> = 80	2.76×10^6^	4.98	5.15×10^5^	6.64
	**Gender**	Female	3.30×10^7^	59.43	4.32×10^6^	55.74
		Male	2.25×10^7^	40.57	3.43×10^6^	44.26
	**Nationality**	Brazilian	5.54×10^7^	99.86	7.73×10^6^	99.84
		Other	7.74×10^4^	0.14	1.23×10^4^	0.16
	**Diagnosis—Top 3**	Spontaneous vertex delivery (O80.0)	4.22×10^6^	7.37	-	-
		Pneumonia, unspecified (J18.9)	1.33×10^6^	2.33	-	-
		Single spontaneous delivery, unspecified (O80.9)	1.16×10^6^	2.03	-	-
		Pure hypercholesterolemia (E78.0)	-	-	5.60×10^5^	7.23
		Sensorineural hearing loss, bilateral (H90.3)	-	-	3.05×10^5^	3.94
		Paranoid schizophrenia (F20.0)	-	-	2.82×10^5^	3.64
	**Procedures—Top 3**	Normal delivery (310010039)	6.11×10^6^	10.66	-	-
		Treatment of pneumonia or influenza (303140151)	4.02×10^6^	7.02	-	-
		Caesarean delivery (411010034)	3.17×10^6^	5.53	-	-
		Phacoemulsification with foldable intraocular lens implantation (405050372)	-	-	5.68×10^5^	7.33
		Cardiac catheterization (211020010)	-	-	4.85×10^5^	6.26
		Evaluation for hearing deficiency diagnosis (211070092)	-	-	4.24×10^5^	5.47

^*^In the hospitalisation table, the number of patients is taken as the number of unique hospitalisation identifiers.

### Representing AIH and APAC datasets using *open*EHR

After the AIH and APAC cleansing process, 61 attributes are represented in the ORBDA source database, from which 20 belong to the AIH table and 47 to the other six APAC tables. We used 19 archetypes rooted in the ENTRY class to model the demographic, administrative and clinical concepts present in the source dataset (Table 3). Three compositions–*demographic_data*, *hospitalisation*, *outpatient_high_complexity_procedures–*were designed to group these concepts into demographic data, and hospitalisation and high complexity procedure authorisations, respectively. Five *ADMIN_ENTRY* archetypes were used to model the administrative and demographic concepts and the clinical events were modelled using 1 *ACTION*, 2 *EVALUATION* and 9 *OBSERVATION* archetypes, containing 63 items, from which 61 belongs to the *ELEMENT* class and 2 to the *CLUSTER* class (Table 4). The *ADMIN_ENTRY* class was used to model administrative concepts, such as admission type and admit date/time, which were found in the admission archetype of the *open*EHR CKM repository. Some other administrative concepts, such as the total stay in ICU and hospitalisation authorisation issue date, were not found in the repository. Thus, a dedicated archetype was created to group the missing hospitalisation authorisation concepts. To model demographic concepts, such as gender and nationality, a new archetype, *demographic_data*, rooted in the *ADMIN_ENTRY* class was created.

**Table 3:** Archetypes and templates used to model the ORBDA dataset.

Archetype	Composition	Template	Type	Source
demographic_data	demographic_data	demographic_data	ADMIN_ENTRY	new
procedure-sus	hospitalisation, outpatient_high_complexity_procedures	bariatrics, chemotherapy, hospitalisation, medication, miscellaneous, nephrology, radiotherapy	ACTION	specialised
admission	hospitalisation	hospitalisation	ADMIN_ENTRY	CKM
hospitalization_authorization	hospitalisation	hospitalisation	ADMIN_ENTRY	new
patient_discharge	hospitalisation, outpatient_high_complexity_procedures	bariatrics, chemotherapy, hospitalisation, medication, miscellaneous, nephrology, radiotherapy	ADMIN_ENTRY	new
problem_diagnosis-sus	hospitalisation, outpatient_high_complexity_procedures	bariatrics, chemotherapy, hospitalisation, medication, miscellaneous, nephrology, radiotherapy	EVALUATION	specialised
high_complexity_procedures_sus	outpatient_high_complexity_procedures	bariatrics, chemotherapy, medication, miscellaneous, nephrology, radiotherapy	ADMIN_ENTRY	new
fluid	outpatient_high_complexity_procedures	nephrology	CLUSTER	CKM
tnm_staging-sus	outpatient_high_complexity_procedures	chemotherapy, radiotherapy	CLUSTER	specialised
bariatric_surgery_evaluation	outpatient_high_complexity_procedures	bariatrics	EVALUATION	new
bodily_output-urination	outpatient_high_complexity_procedures	nephrology	OBSERVATION	CKM
body_mass_index	outpatient_high_complexity_procedures	bariatrics	OBSERVATION	CKM
body_weight	outpatient_high_complexity_procedures	medication, nephrology	OBSERVATION	CKM
height	outpatient_high_complexity_procedures	medication, nephrology	OBSERVATION	CKM
lab_test-antigen_antibody_sus	outpatient_high_complexity_procedures	nephrology	OBSERVATION	specialised
lab_test-blood_glucose	outpatient_high_complexity_procedures	nephrology	OBSERVATION	CKM
lab_test-hba1c	outpatient_high_complexity_procedures	nephrology	OBSERVATION	CKM
lab_test-liver_function	outpatient_high_complexity_procedures	nephrology	OBSERVATION	CKM
lab_test-urea_and_electrolytes-sus	outpatient_high_complexity_procedures	nephrology	OBSERVATION	specialised

**Table 4:** Contents of the ORBDA archetypes.

Archetype	Concept
procedure-sus	**Element**: fields/insertions, irradiated area, procedure, reason/s for procedure, time, vascular access
admission	**Element**: admission type, admit date/time, healthcare unit, hospital service, state/province
demographic_data	**Element**: birth date, educational level, ethnic group, gender, nationality, race
high_complexity_procedures_sus	**Element**: abdominal ultrasonography, age, date of beginning of chemotherapy, date of beginning of radiotherapy, date of first dialysis, duration of treatment, enrolled for transplantation, healthcare unit, indicator of transplantation, issue date, number of transplantations, reason for encounter, schema, state, venous fistula amount
hospitalization_authorization	**Element**: ICU–total, issue date
patient_discharge	**Element**: claim reason, date of discharge, death indicator, hospital infection, reason for discharge
fluid	**Element**: substance, volume
tnm_staging-sus	**Element**: clinical staging, date of pathological identification, histopathological grading, topography
bariatric_surgery_evaluation	**Element**: Baros score, Baros table, follow-up in months
problem_diagnosis-sus	**Element**: associated causes, main diagnosis, regional lymph nodes, secondary diagnosis **Cluster**: staging
bodily_output-urination	**Cluster**: urine detail
body_mass_index	**Element**: body mass index
body_weight	**Element**: weight
height	**Element**: height
lab_test-antigen_antibody_sus	**Element**: HbsAg, HIC—antibodies, HIV
lab_test-blood_glucose	**Element**: glucose
lab_test-hba1c	**Element**: HB
lab_test-liver_function	**Element**: albumin
lab_test-urea_and_electrolytes-sus	**Element**: urea reduction rate

To improve visualisation, prefixes and suffixes of the archetype (including composition) names were removed. The actual name is in the form openEHR-EHR-<TYPE>-<NAME>.v1. For example, for the archetype procedure-sus, the actual archetype name is openEHR-EHR-ACTION-procedure-sus.v1. For the composition name, the TYPE is COMPOSITION, e.g., openEHR-EHR-COMPOSITION-hospitalisation.v1.

Table 4 shows the contents present in each archetype of the ORBDA dataset. Three types of clinical activities were found in the cleaned AIH and APAC datasets: action, observation and evaluation (Table 3). In the *open*EHR model, the *ENTRY* subtype *ACTION* models the information recorded due to the execution of performing an actionable statement, such as a medication order by some agent, and was therefore chosen to represent the procedures undertaken. The CKM *procedure* archetype was specialised to accommodate missing concepts found in the high complexity procedures tables, such as the irradiated area in a chemotherapy procedure. The *OBSERVATION* class is used to record all observed phenomena or state of interest related to the patient, including measurements and pathology results. Thus, it was chosen to model concepts such as height and weight. Essentially, all the observation concepts present in the source dataset could be found in the archetypes of CKM, apart from the antibody and antigens measures, which were specialised in the *lab_test-antigen_antibody_sus* and *lab_test-urea_and_electrolytes-sus* archetypes, respectively. Finally, the EVALUATION class was chosen to model concepts such as diagnosis (main and secondary) and Baros score, as they were all deemed to be assessments or plans made from observations of a procedure.

The statistics of the data types occurrences in the archetypes of the ORBDA dataset are detailed in Table 5. The *open*EHR model is able to express a wide variety of data types (more than 20), allowing the representation of simple Boolean information, e.g., a flag indicating whether a medication was administered or not, to more complex multimedia data, such as medical images. In the ORBDA dataset, 9 data type classes, originated from four packages of the *open*EHR model, are present in the archetypes. Although the data types available in the ORBDA dataset cover less than 50% of the data types available in the *open*EHR model, they are still very representative since they cover most of the common data types available in database management systems, such as string, number (integer and float), date, and boolean.

**Table 5:** Mapped data types found in the archetypes of the ORBDA dataset.

Data type	Package	Occurrence
DV_QUANTITY	quantity	6
DV_BOOLEAN	basic	7
DV_CODED_TEXT	text	23
DV_COUNT	quantity	7
DV_DATE	date time	7
DV_DATE_TIME	date time	3
DV_PROPORTION	quantity	2
DV_TEXT	text	7

### The SUS *open*EHR builder software

**Fig 2:**
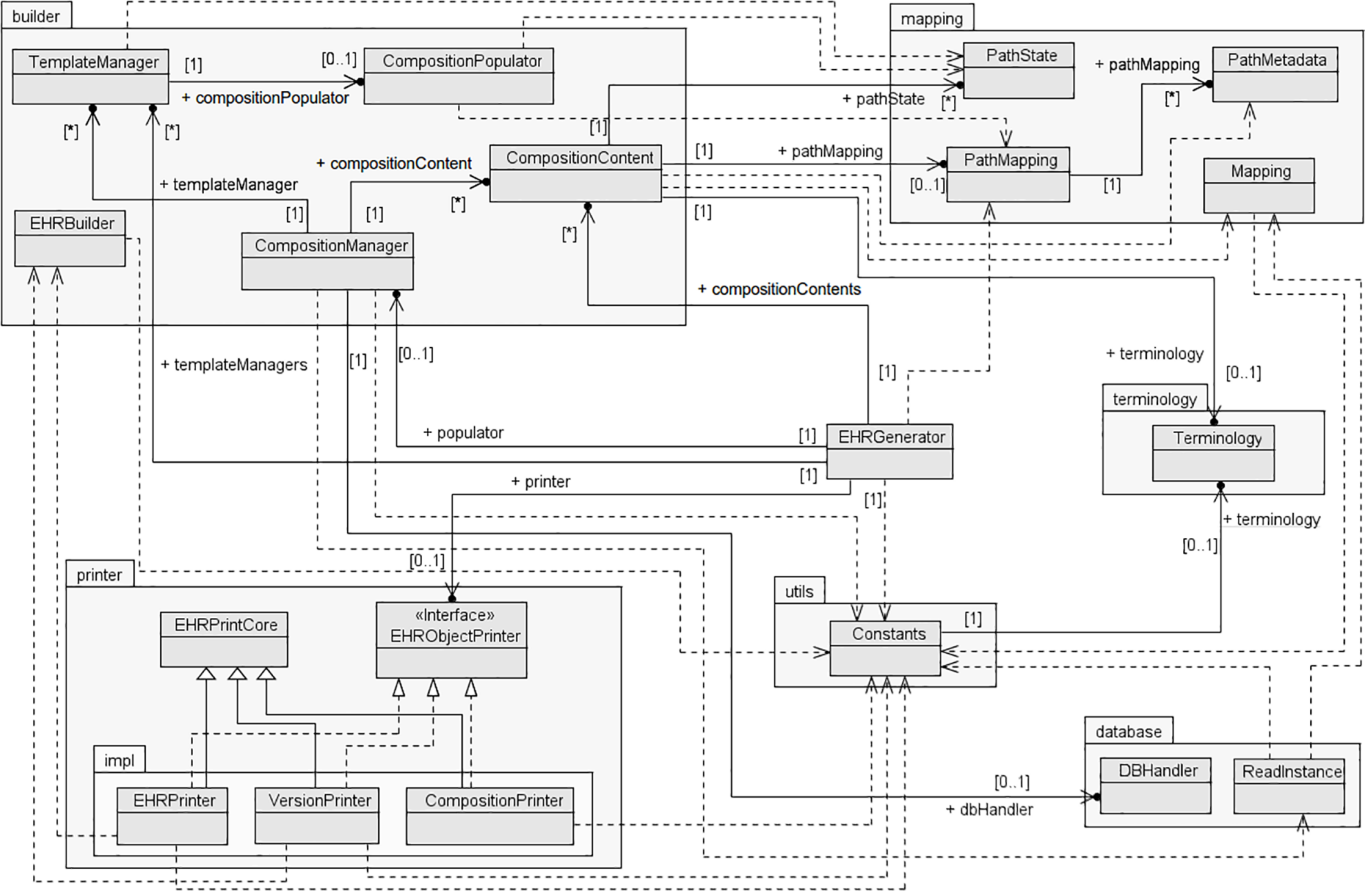
UML class diagram of the SUS-openEHR-Builder software.

The SUS-openEHR-Builder software was developed specifically for generating the ORBDA dataset out of the AIH and APAC relational data. The software creates three types of *open*EHR objects–composition, versioned composition and EHR–in two formats, XML and JSON. To generate these functionalities a Java program following the class diagram of Fig 2 was implemented. The software contains three main packages–database, builder and printer–that are used to extract the data from the relational database, convert it to the *open*EHR format and write it into files in the available formats. At the core of the program, the *open*EHR java-libs library [43] is used to instantiate the *open*EHR elements and serialise them into the XML format. The *open*EHR objects–composition, versioned composition and EHR–are created using the respective classes of the java-libs library. While composition and versioned composition are single, self-contained objects, an EHR is a container object. Each EHR object has zero to several versioned compositions, one EHR status object, which describes some properties of the EHR, such as if it is queriable or modifiable, one EHR access object, which describes the access permissions to the patient EHR, one contribution per versioned composition, one access and status objects, and the EHR container itself. Hence, for each EHR, 5+2×*VC* files are generated, where *VC* is the number of versioned compositions in the EHR. The JSON serialisation is built on top of the XML objects implemented by the java-libs library. Thus, it is expected that JSON objects take longer to be generated. The source code of the SUS-openEHR-Builder software is available at https://github.com/dhteodoro/sus-openehr-builder.

#### Creating ORBDA datasets

Two ORBDA datasets–*all* and *10k* –were created using the SUS-openEHR-Builder software in a parallel setup with 10 jobs to assess the tool and perform the benchmark experiments. The *all* dataset was generated using the whole ORBDA source database (1.5×10^8^ records). It is composed of *open*EHR composition objects in the XML format. In total, it contains 2.1×10^8^ compositions and occupies 2.0 TB of disk space. On the other hand, the *10k* dataset is generated from a subset of the ORBDA source database using 10,000 patient identifiers from the AIH (authorisation ids) and APAC datasets. It is composed of *open*EHR EHR objects and is available in XML and JSON formats. As we can see from Table 6, on average each patient has 2 compositions for the AIH dataset and 13 compositions for the APAC dataset. For the *10k* dataset, we notice that EHR JSON objects are 29% and 33% smaller than EHR XML objects for the AIH and APAC datasets, respectively. To generate the *all* dataset, it took the SUS-openEHR-Builder 6.3 days. AIH XML compositions were created at an average rate of 500 files per second. On the other hand, for APAC XML compositions this rate drops to 313 files per second, still very efficient when compared to literature results [20]. The AIH objects are faster to generate due to their reduced size and archetype complexity. While the size of an AIH XML composition is on average 7.9 KB, an APAC XML composition is 12.4 KB. In addition, APAC objects are more complex in terms of archetype elements (see Table 4), taking slightly longer to be instantiated. As expected, JSON EHR objects are slower to be created compared to XML EHR objects, despite their smaller size. On average, the conversion from XML to JSON adds an overhead of 20% in the EHR object creation time.

**Table 6:** Statistics for the *all* and *10k* datasets created using the SUS-openEHR-Builder.

Dataset	Object	Format	Source	#Patient	#File	Size (GB)	Time (sec)
all	Composition	XML	AIH	55×10^6^	1.1×10^8^	0.85×10^3^	2.2×10^5^
all	Composition	XML	APAC	7.7×10^6^	1.0×10^8^	1.20×10^3^	3.2×10^5^
10k	EHR	XML	AIH	10×10^3^	9.0×10^4^	0.36	1.3×10^2^
10k	EHR	XML	APAC	10×10^3^	3.1×10^5^	2.10	6.7×10^2^
10k	EHR	JSON	AIH	10×10^3^	9.0×10^4^	0.25	1.7×10^2^
10k	EHR	JSON	APAC	10×10^3^	3.1×10^5^	1.40	8.1×10^2^

Statistics for SUS-openEHR-Builder running in parallel setup: 10 jobs.

**Fig 3:**
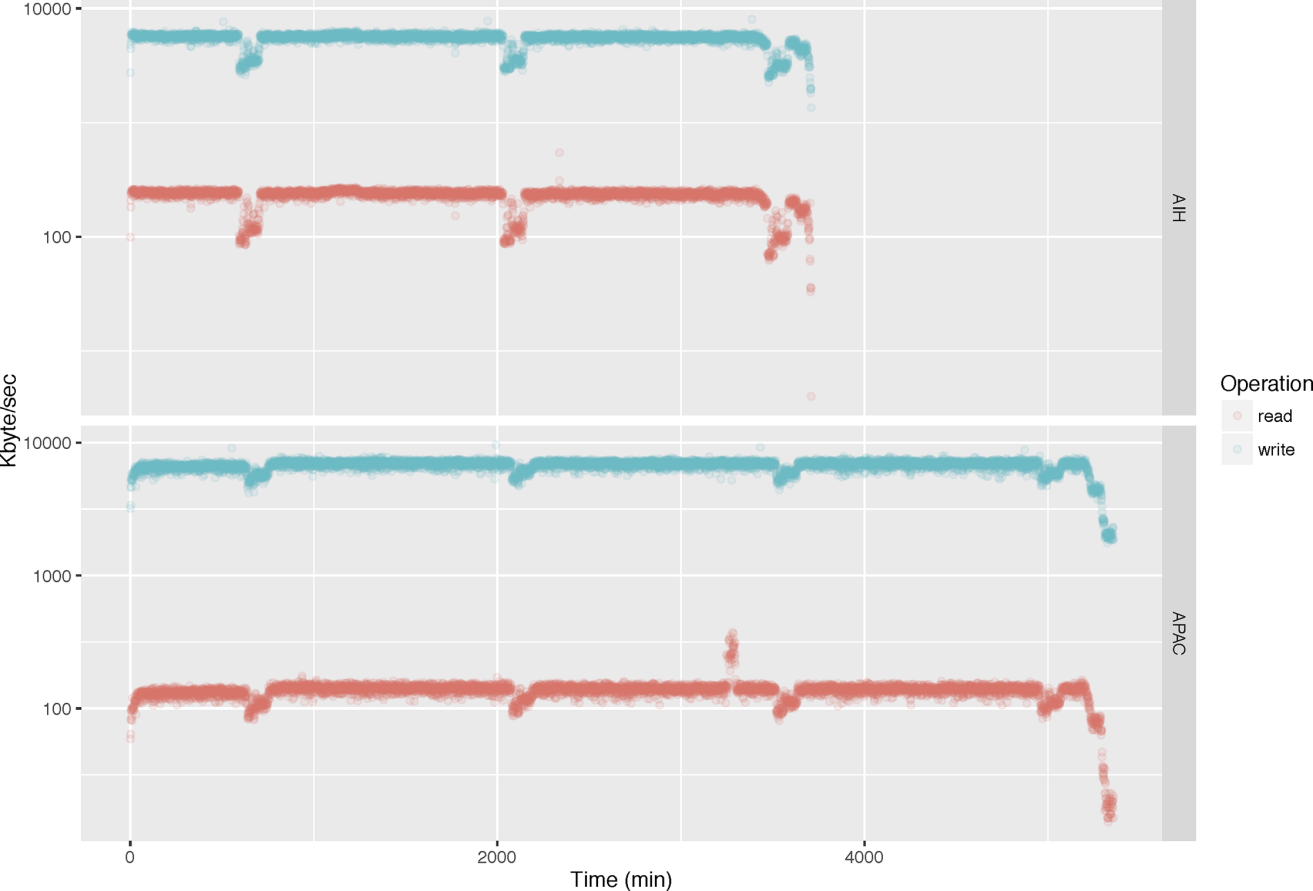
Reading and writing throughput of the SUS-openEHR-Builder tool for generating XML composition objects for the AIH and APAC datasets. 55.47Mi and 7.75Mi patients from the AIH and APAC datasets, respectively (whole ORBDA source database). Parallel setup: 10 jobs.

Fig 3 shows the reading and writing throughput of the SUS-openEHR-Builder to create the *all* collection. While for the AIH dataset data the tool reads from the source database at a median rate of 238 Kbyte/sec (1st Qu.: 225; 3rd Qu.: 247), this rate drops to 138 Kbyte/sec (1st Qu.: 130; 3rd Qu.: 144) for the APAC dataset. This lower rate for the APAC dataset can be explained by the complexity of the query needed to extract data from the six APAC tables. It is also another reason why APAC compositions take longer to be created. Although having a smaller reading rate, the median writing rate of APAC XML compositions is 6854 Kbyte/sec (1st Qu.: 6503; 3rd Qu.: 7099), which is 22% higher than the median writing rate of AIH XML compositions (median: 5607; 1st Qu.: 5345; 3rd Qu.: 5796).

### Benchmark experiments

In this section, we present the results of assessing inserting throughput and retrieval latency for some NoSQL database management systems in different server–client topologies (1 server– 1 client; 1 server– 8 clients; 3 servers– 8 clients) using the ORBDA dataset. We used the *10k* dataset described in the previous section (Table 6), from which the JSON EHR objects were used to test Couchbase and ElasticSearch and the XML EHR objects were used to test eXist-db. Throughput results are showed using three metrics: DOC/sec, EHR/sec and Mbyte/sec. The DOC/sec metric measures the insertion throughput at the document level, where each file of an EHR container is a document. The EHR/sec metric measures the throughput at the EHR container level. Finally, the Mbyte/sec metric measures the throughput from a size perspective. Latency is reported in milliseconds. The 3 servers– 8 clients configuration was not tested for eXist-db due to the lack of native cluster support.

#### Assessing inserting throughput

**Fig 4:**
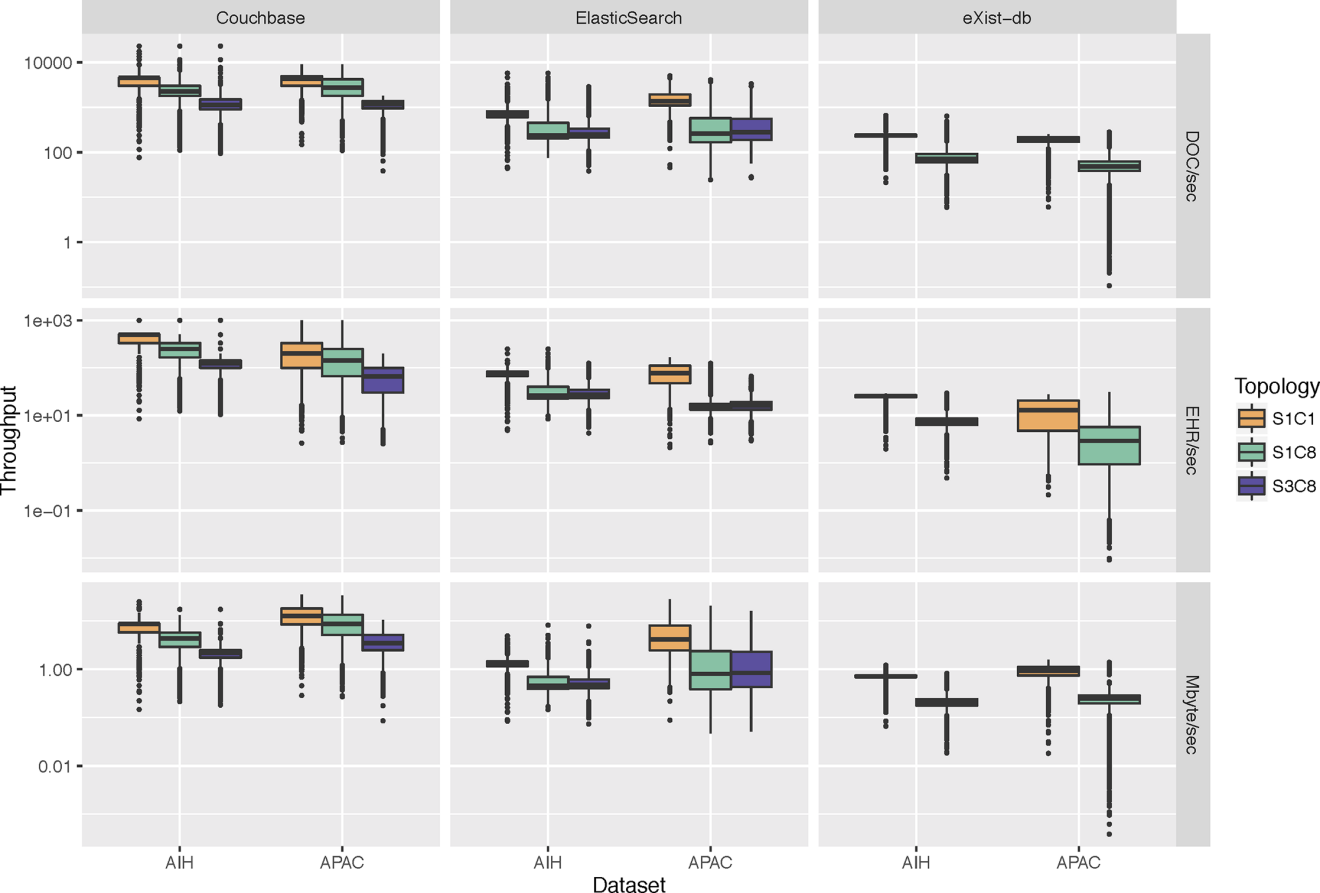
Inserting throughput of Couchbase, ElasticSearch and eXist-db databases in different server–client topologies for the AIH and APAC datasets. 1S1C: 1 server– 1 client; 1S8C: 1 server– 8 clients; 3S8C: 3 servers– 8 clients. EHR in JSON format used in Couchbase and ElasticSearch. EHR in XML format used in eXist-db.

Fig 4 shows the throughput perceived by the client for the DOC/sec, EHR/sec and Mbyte/sec metrics. Overall, Couchbase has the highest inserting throughput for all comparative configurations (*P* < .001). In the 1 server– 1 client topology, the median throughput of Couchbase is 8.69 Mbyte/sec (1st Qu.: 7.21; 3rd Qu.: 14.45) compared to 1.73 Mbyte/sec (1st Qu.: 1.34; 3rd Qu.: 4.14) of ElasticSearch and 0.74 Mbyte/sec (1st Qu.: 0.70; 3rd Qu.: 0.93) of eXist-db. Comparing the inserting performance of the AIH and APAC datasets, in general we notice that for DOC/sec and EHR/sec metrics, the throughput of AIH objects is higher than those of APAC objects (*P* < .001). On the other hand, the throughput for APAC datasets is higher for metric Mbyte/sec (*P* < .001). This holds for all the topologies and database management system apart from the 1 server– 8 clients and 3 servers– 8 clients topologies configuration of Couchbase and ElasticSearch, respectively, where the APAC throughput is also higher than the AIH one for the DOC/sec metric (*P* < .001). As showed in Table 6, on average the size of an APAC composition is larger than an AIH composition and an APAC EHR object has on average 7 times more compositions than an AIH EHR object. Thus, as expected, DOC/sec and EHR/sec metrics for AIH are higher than for APAC objects. However, due to the larger size of APAC objects, its Mbyte/sec throughput tends to be higher.

#### Assessing query latency

**Fig 5:**
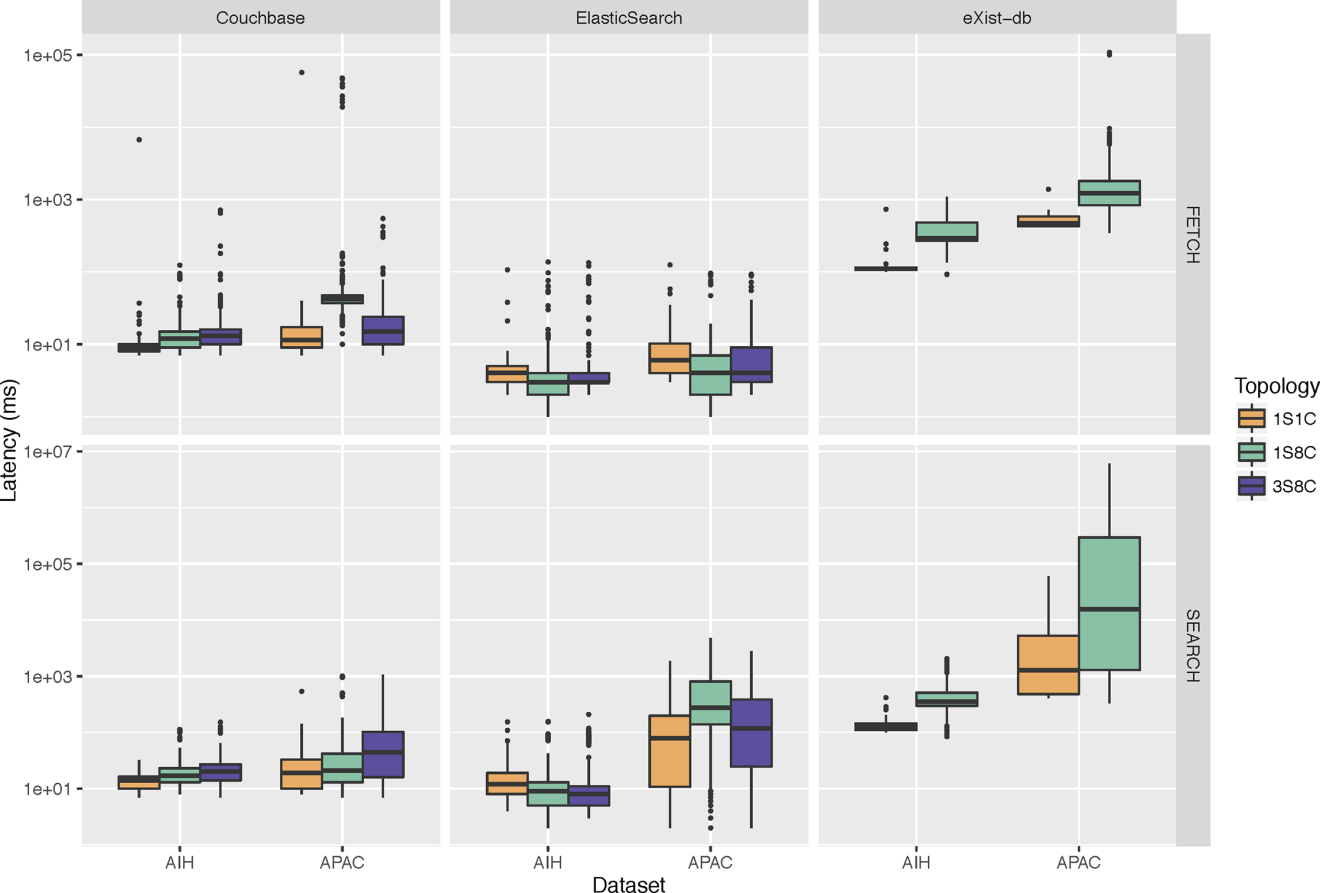
Querying latency of Couchbase, ElasticSearch and eXist-db databases in different server–client topologies for the AIH and APAC datasets. 1S1C: 1 server– 1 client; 1S8C: 1 server– 8 clients; 3S8C: 3 servers– 8 clients. FETCH: search and retrieval of compositions using an EHR identifier. SEARCH: search of EHR identifiers containing a diagnostic code.

To assess the latency of the NoSQL backends storing *open*EHR objects, we created two queries–fetch and search–with 10 variations each. The fetch query retrieves patient data at the individual-level and the search query searches for data at the population-level. The implementation of these queries is showed in the S2 Table and the parameters used are showed in the S1 and S2 Listings. Fig 5 shows the results of the fetch and search queries for the different databases and topologies tested as perceived by the client. Overall, ElasticSearch has the lowest fetch querying latency for all comparative configurations (*P* < .001). In the 1 server– 1 client topology, the median latency of ElasticSearch is 5 ms (1st Qu.: 4; 3rd Qu.: 7) compared to 9 ms (1st Qu.: 8; 3rd Qu.: 14) of Couchbase and up to 416 ms (1st Qu.: 111; 3rd Qu.: 474) of eXist-db. For the search query, the median latency of Couchbase is lower than of ElasticSearch for the 1 server topologies (*P* < .001), while the median latency of ElasticSearch is lower for the 3 servers topology (*P* < .001). More specifically, ElasticSearch outperforms Couchbase for the AIH dataset (*P* < .001), except for the 1 server– 8 clients topology, for which there is no statistically significant difference between them (P = .98). On the other hand, Couchbase outperforms ElasticSearch for the APAC dataset in all topologies (*P* < .001). Both backends outperform eXist-db (*P* < .001), for which latencies are one to two orders of magnitude higher.

With the addition of clients (from 1 to 8), the perceived median latency increases 82% (from 11 ms to 20 ms) and 38% (from 411 ms to 566 ms) for Couchbase and eXist-db backends (*P* < .001), respectively. On the contrary, for ElasticSearch the median latency reduces in 13% (from 8 ms to 7 ms) (*P* < .001). Similarly, the addition of servers (from 1 to 3) has a mixed impact on the query latencies for both Couchbase and ElasticSearch. Overall, the median latency reduces 15% (from 20 ms to 17 ms) for Couchbase (*P* = .02) and 14% (from 7 ms to 6 ms) for ElasticSearch (*P* < .001). This reduction is perceived in the fetch queries against the APAC dataset for Couchbase (64%) and on the search queries for ElasticSearch (54%). However, the median latency is the same for ElasticSearch for the fetch queries against the AIH and APAC datasets (4 ms) and it increases for Couchbase for the search queries (28%).

## Discussion

Systems that adopt multi-level modelling architectures, such as the one proposed by the *open*EHR framework, are supposed to accommodate more easily evolving business needs in comparison with those following standard one-level models [41,47]. Independent of the conceptual model, a keystone step in the engineering of high quality information systems is the testing phase [48]. Studies show that the effort spent in the testing phase is equivalent to the actual system development [49]. In this work, we introduced ORBDA, an *open*EHR benchmark dataset created using a very large real healthcare dataset that can be used to reliably assess and compare persistency mechanisms of *open*EHR-based information systems. Furthermore, we have demonstrated how the ORBDA dataset can be applied to evaluate inserting throughput and query latency of NoSQL backends.

While this paper is focused on *open*EHR, the methods, dataset and conversion software are with some modifications applicable to other similar archetype-based approaches, such as ISO 13606 and CIMI. Different archetype based system implementations all share some central architectural features, semantics and structures, so storage and query approaches working well in one system are likely to work well also in other systems. Archetype based approaches can be used to standardise the semantics and structure of EHR content *inside* EHR systems (not just messages *between* systems). When standardized APIs, like the *open*EHR EHR REST API (www.openehr.org/releases/ITS/latest/ehr_restapi.html), are used in systems, then different storage and query backends can even be interchangeable with fairly limited other EHR system changes. These factors make *shared* research and *comparable* benchmarking of storage and retrieval even more interesting for archetype based systems than for non-standardized EHR systems that instead use unique proprietary models.

### The ORBDA source database and the SUS-openEHR-Builder software

The ORBDA source database is composed of more than 150 million patient records available from the Brazilian Public Health System. ORBDA can be generated in several *open*EHR object and file formats, and sizes thanks to the SUS-openEHR-Builder software. The tool generates *open*EHR objects from DATASUS’ relational data, achieving throughput rates of a few hundreds of files per second. Instead of sharing the benchmark dataset in the *open*EHR format directly, we provide a clean de-identified database, the ORBDA source database, and the tool to convert this relational database into *open*EHR objects. This decision was due to the fact that the whole dataset in the *open*EHR format is too large (*O*(10^12^) bytes), making it difficult for sharing and for users to download it. In addition, this format brings flexibility allowing users to generate datasets with different sizes of load data containing up to *O*(10^6^) patients for the AIH and APAC sets, and to create various types of *open*EHR objects, such as composition and EHR, in different file formats (XML and JSON). Moreover, with some work, SUS-openEHR-Builder can also be extended to serialise *open*EHR objects in other formats, such as Node+Path and Archetype Relational Mapping, as proposed in some relational model configurations [26]. The main drawback is that the dataset is not readily accessible. First, the source database needs to be downloaded and SUS-openEHR-Builder needs to be run to generate the *open*EHR files. To partially mitigate this drawback, we make the *10k* dataset described in this manuscript available at the same address we share the other tools.

As we notice from the benchmarking results, performance tests will have different outcomes depending on whether they use AIH or APAC as the source dataset to generate the ORBDA dataset. The patient identifier in the ORBDA dataset is generated using different semantic identifiers. For the APAC source dataset, it uses the national patient register (CNS). While this identifier is not guaranteed to be unique for a patient due to duplicate registers, it still allows the linkage of several APAC records, resulting in EHR containers with an average of 13 compositions per patient. On the other hand, for the AIH dataset the hospital admission authorization identifier is taken as a surrogate for the patient identifier. Since a new authorization identifier is generated almost for any new patient admission, we have the majority of AIH EHRs with 2 composition records per patient, one demographic and one hospitalisation. It is important to take this idiosyncrasy into account for designing the assessment use cases.

### Representation of ORBDA using archetypes

In the *open*EHR EHR Reference Model, there is no high level grouping class dedicated to model demographics, hospitalisation and high complexity procedure information as found in the AIH and APAC datasets. It would be possible to group parts of an authorisation (hospitalisation or high complexity procedure) under a COMPOSITION or SECTION and create a single archetype for the whole authorisation. However, to follow the organisation of the DATASUS source data, while reducing the redundancy of the demographic information, we created two event COMPOSITION archetypes to represent the hospitalisation and high complexity procedure authorisations and a persistent COMPOSITION was used to organise the demographic data. Instead of using archetypes of the Demographic Model, which are supposed to be stored separately from the other compositions, a new archetype rooted in the ADMIN_ENTRY class was created to model demographic concepts. This decision was taken to simplify storing demographic data, being more usable by existing EHR servers. The Demographic Model of *open*EHR is still under development and not actually implemented neither by many of the *open*EHR tools nor by the servers described in the literature. With the source data and code shared, users of ORBDA will still be able with some effort to represent demographic data using the Demographic Model of *open*EHR if needed.

The strong formalisation and semantics of the *open*EHR model have a price. The most obvious is the increase in size when compared to data oriented formats, such as the relational model. This effect can be easily visualised by the differences between reading (relational model input) and writing (*open*EHR model output) throughput rates in Fig 3. For generating *open*EHR compositions in the XML format, the average difference between reading and writing is 24 and 50 folds for the AIH and APAC datasets, respectively. This fact should be one of the first to be taken into account to properly dimension information systems based on *open*EHR objects.

### Using ORBDA to assess *open*EHR backends

While the main scope of this work was the development and detailed description of the benchmark dataset, we demonstrated how ORBDA could be applied to analyse *open*EHR persistence mechanisms using two main data management operations: writing and reading. In the tests, Couchbase achieved the highest inserting throughput, writing between 10^3^ and 10^4^ *open*EHR documents per second. There was a significant drop in throughput performance in the Couchbase cluster configuration compared to a single server, an issue not verified with ElasticSearch. eXist-db showed a poorer performance compared to the JSON databases, inserting between 10 and 10^3^ documents per second. In the query latency assessment, ElasticSearch had the lowest latency for the *fetch* query types. For the *search* queries, Couchbase had the lowest latency for the APAC dataset and ElasticSearch outperformed Couchbase for the AIH dataset. Similar as in the throughput experiments, eXist-db had the poorest performance, with at least one order of magnitude increased latency. These results are aligned with the existing literature about assessment of *open*EHR persistent mechanisms [25,27]. Notably, the performance of XML databases does not seem suitable for operation in healthcare environments where time constraints are strict. In particular, we notice a significant increase in query latency for eXist-db when adding new clients. To the best of our knowledge, this was the first time parallel client-server operations are reported in *open*EHR assessment.

### Issues and limitations

Despite being based on a real healthcare dataset, it is out of scope for ORBDA to be used in epidemiological studies. While population-based queries, such as the *search* queries presented here, can be realistically exercised, reliable statistics should not be expected from the results. In particular, the use of the hospitalization authorization number as a proxy for the patient identifier in the AIH mischaracterises the dataset from an epidemiological point of view. In addition, for the APAC dataset there is no guarantee that the national patient register is unique for a patient. Moreover, one could be tempted to apply the archetypes and templates designed in this study to test and validate data integration and interoperation architectures for the reuse of clinical data in research. However, we believe that a deeper discussion and validation of the archetypes and templates by experts would be necessary. Therefore, we discourage the use of ORBDA in other contexts than benchmarking assessment.

## Conclusion

In this work, we introduce ORBDA, a publicly available benchmark dataset for evaluation of *open*EHR storage mechanisms. ORBDA is constructed from a very large public healthcare dataset of the Brazilian National Healthcare System containing demographic and clinical information of anonymised in- and outpatients. We provide ORBDA in several *open*EHR formats and dataset sizes using the SUS-openEHR-Builder, a tool that converts relational data into *open*EHR objects. Furthermore, we describe a benchmarking experiment that demonstrates the application of ORBDA for assessing NoSQL data management systems that could also be used as a baseline for testing other *open*EHR databases. We believe that ORBDA is a step forward in performance testing of *open*EHR information systems and therefore could contribute to the engineering, quality improvement and consequent widespread adoption openEHR-based electronic health record systems.

## Supporting information

S1 Table ORBDA source database tables–PostgreSQL datatypes. (DOCX)

S2 Table Parameters used in the query latency assessments. (DOCX)

S1 Listing FETCH query pseudo-code implementation. (DOCX)

S2 Listing SEARCH query pseudo-code implementation. (DOCX)
