# Supplementary material for: ORBDA: An openEHR benchmark dataset for performance assessment of electronic health record servers
Source: PLoS One. 2018 Jan 2;13(1):e0190028. doi: 10.1371/journal.pone.0190028 (PMC5749730; doi:10.1371/journal.pone.0190028)
Supplement: S1 Table — (DOCX) [file pone.0190028.s001.docx]

# S1 Table – ORBDA source database tables – PostgreSQL datatypes

| **Table: hospitalisation** | | | |
| --- | --- | --- | --- |
| **Field** | **Type** | **Key** | **Data element** |
| id_ | serial(10) | PRI | logical primary key |
| n_aih | varchar(1000) | IND | hospitalisation identifier |
| dt_inter | varchar(8) |  | admission date |
| cobranca | varchar(2) |  | claim reason |
| morte | int4(10) |  | death indicator |
| dt_saida | varchar(8) |  | discharge date |
| cgc_hosp | varchar(14) |  | healthcare unit |
| infehosp | varchar(1) |  | hospital infection |
| uti_mes_to | int4(10) |  | ICU – total stay |
| ano_cmpt | varchar(4) |  | issue date |
| mes_cmpt | varchar(2) |  | issue date |
| espec | varchar(2) |  | speciality |
| car_int | varchar(2) |  | type of hospitalisation |
| nasc | varchar(8) |  | birth date |
| instru | varchar(1) |  | educational level |
| sexo | varchar(1) |  | gender |
| nacional | varchar(3) |  | nationality |
| estado | bpchar(2) |  | state |
| diag_princ | varchar(4) |  | main diagnosis |
| proc_rea | varchar(10) |  | performed procedure |
| diag_secun | varchar(4) |  | secondary diagnosis |
| **Table: bariatrics** | | | |
| **Field** | **Type** | **Key** | **Data element** |
| id_ | serial(10) | PRI | logical primary key |
| ap_cnspcn | varchar(1000) | IND | hashed patient identifier |
| ap_dtocor | varchar(8) |  | date of discharge |
| ap_coduni | varchar(7) |  | healthcare unit |
| ap_cmp | varchar(6) |  | issue date |
| ap_motsai | varchar(2) |  | reason for discharge |
| ap_tpaten | varchar(2) |  | reason for encounter |
| ab_dtcirur | varchar(8) |  | date of procedure |
| ab_mesacom | varchar(2) |  | follow-up in months |
| ap_nuidade | varchar(2) |  | age |
| ap_etnia | varchar(4) |  | ethnic group |
| ap_sexo | varchar(1) |  | gender |
| ap_ufnacio | varchar(3) |  | nationality |
| ap_racacor | varchar(2) |  | race |
| estado | bpchar(2) |  | state |
| ap_cidcas | varchar(4) |  | associated causes |
| ap_cidpri | varchar(4) |  | main diagnosis |
| ap_pripal | varchar(10) |  | performed procedure |
| ap_cidsec | varchar(4) |  | secondary diagnosis |
| ab_pontbar | varchar(1) |  | Baros score |
| ab_tabbarr | varchar(1) |  | Baros table |
| ab_imc | varchar(3) |  | body mass index |
| **Table: chemotherapy** | | | |
| **Field** | **Type** | **Key** | **Data element** |
| id_ | serial(10) | PRI | logical primary key |
| ap_cnspcn | varchar(1000) | IND | hashed patient identifier |
| ap_dtocor | varchar(8) |  | date of discharge |
| ap_coduni | varchar(7) |  | healthcare unit |
| ap_cmp | varchar(6) |  | issue date |
| ap_motsai | varchar(2) |  | reason for discharge |
| ap_tpaten | varchar(2) |  | reason for encounter |
| aq_dtintr | varchar(8) |  | date of begin of treatment |
| aq_dtiden | varchar(8) |  | date of pathological identification |
| aq_totmpl | varchar(3) |  | duration of treatment |
| ap_nuidade | varchar(2) |  | age |
| ap_etnia | varchar(4) |  | ethnic group |
| ap_sexo | varchar(1) |  | gender |
| ap_ufnacio | varchar(3) |  | nationality |
| ap_racacor | varchar(2) |  | race |
| estado | bpchar(2) |  | state |
| ap_cidcas | varchar(4) |  | associated causes |
| ap_cidpri | varchar(4) |  | main diagnosis |
| ap_pripal | varchar(10) |  | performed procedure |
| ap_cidsec | varchar(4) |  | secondary diagnosis |
| aq_grahis | varchar(2) |  | histopathological grading |
| aq_linfin | varchar(1) |  | regional lymph nodes |
| aq_esqu_p1 | varchar(5) |  | schema |
| aq_esqu_p2 | varchar(10) |  | schema |
| aq_cid10 | varchar(4) |  | topography |
| aq_estadi | varchar(1) |  | tumour stage |
| **Table: medication** | | | |
| **Field** | **Type** | **Key** | **Data element** |
| id_ | serial(10) | PRI | logical primary key |
| ap_cnspcn | varchar(1000) | IND | hashed patient identifier |
| ap_dtocor | varchar(8) |  | date of discharge |
| ap_coduni | varchar(7) |  | healthcare unit |
| ap_cmp | varchar(6) |  | issue date |
| ap_motsai | varchar(2) |  | reason for discharge |
| ap_tpaten | varchar(2) |  | reason for encounter |
| am_transpl | varchar(1) |  | indicator of transplantation |
| am_qtdtran | varchar(2) |  | number of transplantations |
| ap_nuidade | varchar(2) |  | age |
| ap_etnia | varchar(4) |  | ethnic group |
| ap_sexo | varchar(1) |  | gender |
| ap_ufnacio | varchar(3) |  | nationality |
| ap_racacor | varchar(2) |  | race |
| estado | bpchar(2) |  | state |
| ap_cidcas | varchar(4) |  | associated causes |
| ap_cidpri | varchar(4) |  | main diagnosis |
| ap_pripal | varchar(10) |  | performed procedure |
| ap_cidsec | varchar(4) |  | secondary diagnosis |
| am_altura | varchar(3) |  | height |
| am_peso | varchar(3) |  | weight |
| **Table: miscellaneous** | | | |
| **Field** | **Type** | **Key** | **Data element** |
| id_ | serial(10) | PRI | logical primary key |
| ap_cnspcn | varchar(1000) | IND | hashed patient identifier |
| ap_dtocor | varchar(8) |  | date of discharge |
| ap_coduni | varchar(7) |  | healthcare unit |
| ap_cmp | varchar(6) |  | issue date |
| ap_motsai | varchar(2) |  | reason for discharge |
| ap_tpaten | varchar(2) |  | reason for encounter |
| ap_nuidade | varchar(2) |  | age |
| ap_etnia | varchar(4) |  | ethnic group |
| ap_sexo | varchar(1) |  | gender |
| ap_ufnacio | varchar(3) |  | nationality |
| ap_racacor | varchar(2) |  | race |
| estado | bpchar(2) |  | state |
| ap_cidcas | varchar(4) |  | associated causes |
| ap_cidpri | varchar(4) |  | main diagnosis |
| ap_pripal | varchar(10) |  | performed procedure |
| ap_cidsec | varchar(4) |  | secondary diagnosis |
| **Table: nephrology** | | | |
| **Field** | **Type** | **Key** | **Data element** |
| id_ | serial(10) | PRI | logical primary key |
| ap_cnspcn | varchar(1000) | IND | hashed patient identifier |
| ap_dtocor | varchar(8) |  | date of discharge |
| ap_coduni | varchar(7) |  | healthcare unit |
| ap_cmp | varchar(6) |  | issue date |
| ap_motsai | varchar(2) |  | reason for discharge |
| ap_tpaten | varchar(2) |  | reason for encounter |
| an_ulsoab | varchar(1) |  | abdominal ultrasonography |
| an_dtpdr | varchar(8) |  | date of first dialysis |
| an_cncdo | varchar(1) |  | enrolled for transplantation |
| ap_nuidade | varchar(2) |  | age |
| ap_etnia | varchar(4) |  | ethnic group |
| ap_sexo | varchar(1) |  | gender |
| ap_ufnacio | varchar(3) |  | nationality |
| ap_racacor | varchar(2) |  | race |
| estado | bpchar(2) |  | state |
| ap_cidpri | varchar(4) |  | main diagnosis |
| ap_pripal | varchar(10) |  | performed procedure |
| ap_cidsec | varchar(4) |  | secondary diagnosis |
| an_albumi | varchar(2) |  | albumin |
| an_diures | varchar(4) |  | diuresis |
| an_glicos | varchar(4) |  | glucose |
| an_hb | varchar(2) |  | HB |
| an_hbsag | varchar(1) |  | HbsAg |
| an_altura | varchar(3) |  | height |
| an_hcv | varchar(1) |  | HIC – antibodies |
| an_hiv | varchar(1) |  | HIV |
| an_tru | varchar(4) |  | urea reduction rate |
| an_acevas | varchar(1) |  | vascular access |
| an_intfis | varchar(2) |  | venous fistula amount |
| an_peso | varchar(3) |  | weight |
| **Table: radiotherapy** | | | |
| **Field** | **Type** | **Key** | **Data element** |
| id_ | serial(10) | PRI | logical primary key |
| ap_cnspcn | varchar(1000) | IND | hashed patient identifier |
| ap_dtocor | varchar(8) |  | date of discharge |
| ap_coduni | varchar(7) |  | healthcare unit |
| ap_cmp | varchar(6) |  | issue date |
| ap_motsai | varchar(2) |  | reason for discharge |
| ap_tpaten | varchar(2) |  | reason for encounter |
| ar_dtintr | varchar(8) |  | date of begin of treatment |
| ar_dtiden | varchar(8) |  | date of pathological identification |
| ar_finali | varchar(1) |  | reason for treatment |
| ap_nuidade | varchar(2) |  | age |
| ap_etnia | varchar(4) |  | ethnic group |
| ap_sexo | varchar(1) |  | gender |
| ap_ufnacio | varchar(3) |  | nationality |
| ap_racacor | varchar(2) |  | race |
| estado | bpchar(2) |  | state |
| ap_cidcas | varchar(4) |  | associated causes |
| ap_cidpri | varchar(4) |  | main diagnosis |
| ap_pripal | varchar(10) |  | performed procedure |
| ap_cidsec | varchar(4) |  | secondary diagnosis |
| ar_grahis | varchar(2) |  | histopathological grading |
| ar_cidtr1 | varchar(4) |  | irradiated area |
| ar_cidtr2 | varchar(4) |  | irradiated area |
| ar_cidtr3 | varchar(4) |  | irradiated area |
| ar_numc1 | varchar(3) |  | number of fields/insertions |
| ar_numc2 | varchar(3) |  | number of fields/insertions |
| ar_numc3 | varchar(3) |  | number of fields/insertions |
| ar_linfin | varchar(1) |  | regional lymph nodes |
| ar_cid10 | varchar(4) |  | topography |
| ar_estadi | varchar(1) |  | tumour stage |
