# Supplementary material for: ORBDA: An openEHR benchmark dataset for performance assessment of electronic health record servers
Source: PLoS One. 2018 Jan 2;13(1):e0190028. doi: 10.1371/journal.pone.0190028 (PMC5749730; doi:10.1371/journal.pone.0190028)
Supplement: S2 Table — (DOCX) [file pone.0190028.s002.docx]

# S2 Table – Parameters used in the query latency assessments

| **Query id** | **Dataset** | **FETCH parameter** | **SEARCH parameter** |
| --- | --- | --- | --- |
| Q1 | AIH | 1.1.1.9798575750565.4485556539951.9857102575349.9910099505010.0551009953505654 | A90 |
| Q2 |  | 1.1.1.5053514897975.5979810152499.9545456100535.2535399551005.651524849579749 | N47 |
| Q3 |  | 1.1.1.57102989997575.55297545648481.02561019899991.00569950100519.752521019955102 | L031 |
| Q4 |  | 1.1.1.5157574950529.8534898504810.2549952541029.7564953994999.57525650544853 | O420 |
| Q5 |  | 1.1.1.57101485410050.51515110254995.09810252545249.55499897100100.54515297494855 | O808 |
| Q6 |  | 1.1.1.49999951100101.55485498495310.09810048994950.10153971005198.9855561019710053 | J189 |
| Q7 |  | 1.1.1.10049535510053.54551019954575.05099499910210.09956574997544.84951535457102 | I64 |
| Q8 |  | 1.1.1.53531001011014.81024956491025.29752995648985.65551100535399.51985310153100100 | J158 |
| Q9 |  | 1.1.1.5299995751541.0148521025551.1025749999710.1519910253575.25257525455995497 | F200 |
| Q10 |  | 1.1.1.55549948981015.25654100559752.54100515310048.51102101525398.5355971011015649 | E149 |
| Q1 | APAC | 1.1.1.489752100565248525010049995.050100499949505354999854545.653101495757975110149555451.559853101505456494910297974.85750971015254994851555153101 | E788 |
| Q2 |  | 1.1.1.4897521005652485250100499950.5010049102565698545110249985.2985554979910097975153555797.5310152979948501005310153515.51001025057525210057495210049 | C504 |
| Q3 |  | 1.1.1.4897521005652485250100499950.5010049989953495598985051991.0099564848499853511019998545.0524810110199565653555555525.25551101101100101991021005051 | C675 |
| Q4 |  | 1.1.1.489752100565248525010049995050100.499854524956545554549710150995655.481024852995457554849525352501015.650575698494855995256545550495754.1015251575352521025397505698499810150 | F209 |
| Q5 |  | 1.1.1.4897521005652485250100499950.5010049101101100559998501001.0252549799541029910049989849.9856100505451535555979853529.99849561001019850981001005710050 | M815 |
| Q6 |  | 1.1.1.4897521005652485250100499950.5010049100544810098541005449.1005110251101989799549752975.3995510157535610153561005097.5553102975757495510055101575755 | F900 |
| Q7 |  | 1.1.1.489752100565248525010049995.050100494855995352991004951.484853541025348511025310098.525753575651999750561009755.995010054971025457499848565152 | C12 |
| Q8 |  | 1.1.1.1024956101485510255515210257.5210249501024810110154995250.5354525110110054555099971019.7989752551014951985797989952.10249102575156100505153579910248 | M810 |
| Q9 |  | 1.1.1.4897521005652485250100499950.5010049564910250994957555797.5055565051489856100555710253.4951494952102481014910098995.5102102491005049579899574853 | F200 |
| Q10 |  | 1.1.1.489752100565248525010049995.050100499798559799984948545.098495052535749549956102535.350519751989899101499910048.50551025510151541005453494853 | Z940 |
