# Supplementary material for: ORBDA: An openEHR benchmark dataset for performance assessment of electronic health record servers
Source: PLoS One. 2018 Jan 2;13(1):e0190028. doi: 10.1371/journal.pone.0190028 (PMC5749730; doi:10.1371/journal.pone.0190028)
Supplement: S1 Listing — (DOCX) [file pone.0190028.s003.docx]

# S1 Listing - FETCH query pseudo-code implementation

**Couchbase**

bucket.query(ViewQuery.from("tests", "by_ehr_id").stale(Stale.FALSE).key(ehr_id))

view := " by_ehr_id "

function (doc, meta) {

if(doc.versions.data) {

if(doc.versions.data.type == "COMPOSITION"); {

emit(doc.owner_id.id.value, doc.versions.data);

} } }

**ElasticSearch**

client.prepareSearch(index)

.setTypes(type).setScroll(new TimeValue(60000))

.setQuery(QueryBuilders.boolQuery()

.must(QueryBuilders.termQuery("owner_id.id.value", ehr_id))

.must(QueryBuilders.termQuery("versions.data.type", "COMPOSITION")))

.setSize(scrollSize).execute().actionGet()

**eXist-db**

runQuery(“xquery version '3.0'

declare default element namespace 'http://schemas.openehr.org/v1';

for $match in collection('/db/ORBDA')//versioned_composition/owner_id/id[value = '"+ ehr_id +"']

return <versions>{$match/ancestor::*/versions/data}</versions>”

)
