# Supplementary material for: ORBDA: An openEHR benchmark dataset for performance assessment of electronic health record servers
Source: PLoS One. 2018 Jan 2;13(1):e0190028. doi: 10.1371/journal.pone.0190028 (PMC5749730; doi:10.1371/journal.pone.0190028)
Supplement: S2 Listing — (DOCX) [file pone.0190028.s004.docx]

# S2 Listing - SEARCH query pseudo-code implementation

**Couchbase**

bucket.query(ViewQuery.from("tests", "by_icd").stale(Stale.FALSE).key(icd_code))

view := "by_icd"

function (doc, meta) {

if(doc.versions.data.type == "COMPOSITION") {

for(var i=0; i<doc.versions.data.content.length; i++) {

if(doc.versions.data.content[i].archetype_node_id == "openEHR-EHR-EVALUATION.problem_diagnosis-sus.v1") {

for(var j=0; j<doc.versions.data.content[i].data.items.length; j++) {

if(doc.versions.data.content[i].data.items[j].archetype_node_id == "at0002.1") { emit(doc.versions.data.content[i].data.items[j].value.defining_code.code_string,doc.owner_id.id.value);

} } } } } }

**ElasticSearch**

client.prepareSearch(index)

.setTypes(type).setScroll(new TimeValue(60000))

.setFetchSource(new String[]{"owner_id.id.value"}, null)

.setQuery(QueryBuilders.boolQuery() .filter(QueryBuilders.termQuery("versions.data.content.data.items.value.defining_code.code_string", icd_code))

.filter(QueryBuilders.termQuery("versions.data.content.archetype_node_id", "openEHR-EHR-EVALUATION.problem_diagnosis-sus.v1")))

.setSize(scrollSize).execute().actionGet()

**eXist-db**

runQuery(“xquery version '3.0'

declare namespace v1 = 'http://schemas.openehr.org/v1'

declare default element namespace 'http://schemas.openehr.org/v1'

declare namespace xsi = 'http://www.w3.org/2001/XMLSchema-instance'

for $match in collection('/db/ORBDA')//versioned_composition//*[code_string = '" + icd_code + "']

return <value>{$match/ancestor::*/owner_id/id/value/text()}</value>”

)
